# A wearable sensor and machine learning estimate step length in older adults and patients with neurological disorders

**DOI:** 10.1038/s41746-024-01136-2

**Published:** 2024-05-25

**Authors:** Assaf Zadka, Neta Rabin, Eran Gazit, Anat Mirelman, Alice Nieuwboer, Lynn Rochester, Silvia Del Din, Elisa Pelosin, Laura Avanzino, Bastiaan R. Bloem, Ugo Della Croce, Andrea Cereatti, Jeffrey M. Hausdorff

**Affiliations:** 1 Center for the Study of Movement, Cognition and Mobility, Neurological Institute, Tel Aviv Medical Center, Tel Aviv, Israel; 2 Department of Biomedical Engineering, Faculty of Engineering, Tel Aviv University, Tel Aviv, Israel; 3 Department of Industrial Engineering, Faculty of Engineering, Tel Aviv University, Tel Aviv, Israel; 4 Faculty of Medical & Health Sciences and Sagol School of Neuroscience, Tel Aviv University, Tel Aviv, Israel; 5 Department of Rehabilitation Science, KU Leuven, Neuromotor Rehabilitation Research Group, Leuven, Belgium; 6 Translational and Clinical Research Institute, Faculty of Medical Sciences, Newcastle University, Tyne, NE1 7RU UK; 7 National Institute for Health and Care Research (NIHR) Newcastle Biomedical Research Centre (BRC), Newcastle University and The Newcastle upon Tyne Hospitals NHS Foundation Trust, Newcastle upon Tyne, UK; 8 Department of Neuroscience, Rehabilitation, Ophthalmology, Genetics and Maternal Child Health (DINOGMI), University of Genoa, Genoa, Italy; 9 IRCCS Policlinico San Martino Teaching Hospital, Genoa, Italy; 10 Department of Experimental Medicine, Section of Human Physiology, University of Genoa, Genoa, Italy; 11 Radboud university medical center, Donders Institute for Brain, Cognition, and Behavior; Department of Neurology, Nijmegen, The Netherlands; 12 Department of Biomedical Sciences, University of Sassari, Sassari, Italy; 13 Department of Electronics and Telecommunications, Politecnico di Torino, Turin, Italy; 14 Department of Physical Therapy, Faculty of Medicine, Tel Aviv University, Tel Aviv, Israel; 15 Department of Orthopedic Surgery, Rush Alzheimer’s Disease Center and Rush University Medical Center, Chicago, Illinois USA

**Keywords:** Diagnostic markers, Neurological disorders

## Abstract

Step length is an important diagnostic and prognostic measure of health and disease. Wearable devices can estimate step length continuously (e.g., in clinic or real-world settings), however, the accuracy of current estimation methods is not yet optimal. We developed machine-learning models to estimate step length based on data derived from a single lower-back inertial measurement unit worn by 472 young and older adults with different neurological conditions, including Parkinson’s disease and healthy controls. Studying more than 80,000 steps, the best model showed high accuracy for a single step (root mean square error, RMSE = 6.08 cm, ICC(2,1) = 0.89) and higher accuracy when averaged over ten consecutive steps (RMSE = 4.79 cm, ICC(2,1) = 0.93), successfully reaching the predefined goal of an RMSE below 5 cm (often considered the minimal-clinically-important-difference). Combining machine-learning with a single, wearable sensor generates accurate step length measures, even in patients with neurologic disease. Additional research may be needed to further reduce the errors in certain conditions.

## Introduction

Step length is generally reduced with aging^1,2^ and among people with neurological disorders^3,4^. The gait cycle represents a series of movements repeated in a walking pattern^5^. A step refers to one single step during the cycle, while a stride refers to an entire cycle; since a single stride consists of two steps, step length and stride length are typically highly correlated. Both of these spatial measures of gait, i.e., step length and stride length, are also highly correlated with gait speed^6^. Indeed, in studies that have grouped the spatial-temporal parameters of gait into different domains (for example, via principal component analyses), it is now relatively common to refer to pace (e.g., step length, gait speed), rhythm (e.g., cadence), and variability (e.g., step-to-step changes in step length)^7,8^. Alterations in these key spatial-temporal measures of gait, especially step length, predict adverse health outcomes such as falls, cognitive decline, dementia, morbidity, mortality^6,9,10^, and the response to interventions^4,11^. Given its importance and ability to reflect aging and the disease stage (e.g., in Parkinson’s disease), step length has also been used as an outcome measure^12–15^. While large changes in step length can be observed visually, quantitative estimations are required to accurately determine subtle changes in step length over time, monitor the response to therapy, and evaluate disease progression^16^. This ability can allow for better assessment of changes associated with aging, improve the capacity to objectively detect and track disease, and enhance the ability to quantify the impact of interventions^16,17^.

Conventional methods for obtaining step length estimation (SLE) include camera-based systems and instrumented gait mats. These methods are accurate; however, they only provide a snapshot view of a person’s walking at a given instant in time. These snapshot observations may be biased by many factors, such as the time of the day, medication, affect, and white coat syndrome^18–21^. Indeed, a growing body of literature suggests that continuous (e.g., 24/7) monitoring of gait is clinically meaningful and that it captures information that cannot be measured by conventional test of walking ability in the clinic or lab (such as variations in the gait pattern across the week)^1,18–27^. Moreover, continuous monitoring bridges the gap between measures of gait taken during daily living and those taken in a lab^1,22–24^. The latter may reflect capacity, while the former captures actual, real-world function. To optimally characterize the gait of an older adult and patients with neurological diseases, it may, therefore, be helpful to measure gait over an extended period of time^25–27^ Camera-based systems and instrumented gait mats cannot be used for that purpose but inertial measurement units have the potential to meet that goal.

A 3D inertial measurement unit (IMU) is an electronic device that measures accelerations and angular velocities in three perpendicular directions. With a wearable design, IMUs are lightweight and relatively inexpensive and, therefore, can be incorporated into gear such as smartwatches, shoe insoles, or dedicated sensors placed at different locations on the body. Thus, IMUs can be applied in clinical settings and also leveraged to assess real-world walking over an extended period of time. Using a wearable device mounted on the lower back, acquired IMU signals can estimate and analyze gait parameters, including step length^28,29^. However, since IMUs do not directly measure spatial parameters, an estimator or model is required.

In general, three different approaches have been used to estimate step length from an IMU. These are double integration^30^, kinematic human gait modeling^31^, and regression methods^31^. The double integration of accelerometer data involves sequentially integrating acceleration to derive velocity and then integrating velocity to estimate displacement, providing a method to assess step length in gait analysis^31^. However, the result tends to drift over time^32^ or requires using zero velocity updates (ZUPT) that are effective only if the IMU is placed near the foot^33^. The kinematic human gait modeling option usually performs better with calibration^31^, limiting its widespread application. Kose et al. ^34^ estimated step length using a combination of a Kalman filter and an optimally filtered direct and reverse integration applied to the IMU signals.

In recent years, with the rapid development of machine learning (ML), researchers have aimed to develop a SLE regression model^31,35^. In several studies^36,37^, the investigators tried to estimate the step length and walking speed, respectively, using data acquired from a smartwatch. After preprocessing that included filtering and segmenting the steps, a variety of machine and deep learning models were attempted, including Linear Regression (LR), Gaussian Process Regression (GPR), Support Vector Machine (SVM), Regression Tree (RT), convolutional neural network (CNN), and least short-term memory (LSTM). Although these studies^34,36,37^ demonstrated high potential for SLE and walking speed estimation in the specified dataset, the generalizability was restricted because the models were derived from a small training dataset that included only young and healthy participants.

To address this gap, Byun et al. conducted a study on older adults^38^. An IMU containing a 3D accelerometer and 3D gyroscope was located on the lower back, approximately at the height of the center of mass. The model was improved by applying a slow speed-specific regression model sequentially after the estimation of gait speed by a general regression model. The proposed method achieved relatively good estimation accuracy for gait speed with a root mean squared error (RMSE) of 6.81 ${cm}/s$. However, in addition to the features extracted from the IMU, the researchers used demographic and anthropometric features that required a large number of manual measurements and frequent calibration (e.g., if the subject’s weight changes over time). The study by Hannink et al. ^39^ also included a relatively large number (*n* = 116) of older adults. The IMUs were placed laterally below each ankle joint. Using a convolutional neural network (CNN), an accurate estimate of the stride length (RMSE = 6.09 ${cm}$) was obtained. The chosen location for the IMU was the main disadvantage in this study due to the unconventional location for the body worn device, which can negatively affect compliance.

A more recent study from the Mobilise-D consortium^40^ focusing on patient groups (e.g., Parkinson’s disease, multiple sclerosis) and older adults assessed and validated stride length estimators on 108 participants from six different cohorts. The participants were monitored for about 2.5 h during the day as they conducted routine daily living activities. The absolute error between the estimations of stride length and the reference system ranged from 15 to 33 cm across all algorithms. These results are impressive considering the challenging settings of everyday activities and the health status and age of the cohort. Nevertheless, ideally, further reduction in the error would be useful for reliably detecting even relatively small changes in step length.

The Minimal-Clinically Important-Difference (MCID) is an important concept in clinical research. It represents the smallest change in a variable of interest that is considered clinically meaningful, signifying a perceptible change in the patient’s condition or meaningful effectiveness of an intervention^41,42^. Prior research indicates that the MCID of gait speed for adults with diverse health conditions, such as multiple sclerosis (MS), acute cardiovascular disease, and stroke, typically falls within the range of 10 to 20 cm/s^43,44^. Taking into consideration the average duration of typical steps (~0.5 s)^45,46^, this gait speed MCID translates to a step length MCID of 5 cm. A more recent paper showed that the MCID for individuals with Parkinson’s disease (PD) is 3.6 cm^47^. Therefore, to estimate the step length accurately and continuously, a more generalizable model with a wearable device located in a convenient location is still needed. To this end, we leveraged previously collected data to generate a relatively large (*n* = 472) and diverse set of data, comprising five different groups of participants with a range of health status and gait abilities: individuals with PD, those with mild cognitive impairment (MCI), individuals with MS, healthy young, and older adults. Theoretically, the use of this diverse dataset can contribute to the generalizability of the model and enable a more comprehensive SLE analysis across different populations; this goal of generalizability and using a single model in diverse cohorts is shared by an approach that was also taken in the Mobilise-D study^25^ as it potentially allows for more widespread application.

The goal of the present work is to develop a regression model that can estimate the step length more accurately than current solutions (below 5 cm) using IMU data collected from a single lower back wearable device during a straightline walking trajectory in a laboratory setting. The study’s primary contribution lies in the creation of a generalized model, trained and tested on diverse populations, that estimates step length accurately in older adults and people with neurological disease, without the need for calibration or the use of any demographic or anthropometric features. Furthermore, for some purposes, it may be beneficial to calculate the average over several steps to achieve a more reliable SLE. Indeed, when considering the pace domain (i.e., step length), one very common practice is to average multiple steps taken in a walk in order to provide a single, representative summary of this gait parameter. This averaging approach is commonly used during in-lab and clinic studies of gait; the walking test typically lasts between 30 s to 6 min and the average is used to describe this feature^13,48^. This averaging technique can reduce noise that may affect the computed features used to estimate the step length. Therefore, considering the average over several steps provides a single and more robust representation for estimating the patient’s gait parameters. The trade-off between the SLE accuracy obtained by this method and the ability to estimate instantaneous step length was also explored. Assessing this trade-off is important for outcomes such as step-to-step variability that can provide additional diagnostic, prognostic, and mechanistic information^13,49,50^ when instantaneous values of step length are available and step-to-step variability can be determined. In addition, we also aimed to better understand the results of the models in terms of the feature importance, error analysis, and the impact of gait speed, which can be used for future improvements.

## Results

### Subject demographics and description of the train and test sets

Age, height, and step length of the participants, as measured by the Zeno Walkway, are presented in Table 1 as means and standard deviation, whereas gender is presented as the percentage of females from the total number of participants. A relatively wide range of values is seen. The collected data for model training and initial testing includes three participant groups—people with Parkinson’s disease (PD), subjects with mild cognitive impairment (MCI), and a group of older adults (OA). Additionally, the table presents the way the data was split for the ML experiments. The top part of the table describes the main dataset that was used to train and test the model. It is denoted as the “test set”. The other two datasets (central and bottom parts of the table) serve as out-of-distribution examples. These include people with multiple sclerosis, an age-matched healthy control group, people with PD, and age-matched healthy control older adults. Their purpose is to verify the generalization abilities of the constructed model to process and predict data that were gathered from a slightly different population; we refer to these datasets as the validation set. Evaluation of the model for this type of out-of-distribution data is one of the main contributions of the work, showcasing the robustness of the selected ML model and it related features.

**Table 1:** Demographic and clinical characteristics of the study participants

Characteristic	Age (years)	Gender: %females	Height (m)	Weight (kg)	Step length (cm)				Gait speed (cm/s)			
					Overall	Usual	Fast	Dual tasking	Overall	Usual	Fast	Dual tasking
Test Set (V-TIME participants)												
Overall (*n* = 257)	74.03 ± 6.73	54.68	1.66 ± 0.09	71.57 ± 13.69	57.72 ± 11.23	56.63 ± 10.15	62.17 ± 11.05	53.97 ± 10.86	109.98 ± 28.76	104.55 ± 23.25	126.58 ± 29.19	97.39 ± 24.64
Per group												
OA (*n* = 81)	76.46 ± 6.30	78.65	1.63 ± 0.09	68.96 ± 13.59	58.94 ± 11.10	57.53 ± 10.17	63.17 ± 11.08	55.65 ± 10.55	114.24 ± 29.10	107.15 ± 22.77	131.68 ± 29.86	101.97 ± 24.29
MCI (*n* = 27)	78.37 ± 6.19	75.00	1.63 ± 0.07	69.66 ± 13.52	56.94 ± 11.70	55.81 ± 10.78	60.78 ± 12.27	53.73 ± 10.70	108.10 ± 31.95	102.35 ± 27.44	122.72 ± 33.87	97.49 ± 27.78
PD (*n* = 149)	71.77 ± 6.19	36.94	1.68 ± 0.09	73.44 ± 13.62	56.95 ± 11.20	55.98 ± 10.07	61.49 ± 10.85	53.02 ± 10.93	107.41 ± 27.61	102.64 ± 22.63	123.51 ± 27.47	94.88 ± 23.84
**Validation set 1**												
Overall (*n* = 113)	68.35 ± 7.77	40.71	1.68 ± 0.09	75.14 ± 12.55	58.85 ± 9.83	n/a	n/a	n/a	101.06 ± 27.26	n/a	n/a	n/a
Per group												
OA (*n* = 38)	69.07 ± 8.71	44.74	1.67 ± 0.10	75.57 ± 13.85	64.21 ± 6.97	n/a	n/a	n/a	115.84 ± 22.56	n/a	n/a	n/a
PD (*n* = 75)	67.98 ± 7.25	38.67	1.69 ± 0.09	74.93 ± 11.93	54.71 ± 9.22	n/a	n/a	n/a	90.59 ± 26.21	n/a	n/a	n/a
**Validation set 2**												
Overall (*n* = 102)	40.00 ± 11.13	60.78	1.69 ± 0.09	70.40 ± 15.38	73.94 ± 12.69	n/a	n/a	n/a	144.16 ± 39.01	n/a	n/a	n/a
Per group												
HA (*n* = 41)	37.03 ± 10.32	51.22	1.70 ± 0.09	70.93 ± 13.57	79.03 ± 9.85	n/a	n/a	n/a	163.52 ± 31.69	n/a	n/a	n/a
MS (*n* = 61)	42.00 ± 11.29	67.21	1.69 ± 0.10	70.04 ± 16.59	68.48 ± 12.69	n/a	n/a	n/a	130.01 ± 37.76	n/a	n/a	n/a

Values are presented as mean ± standard deviation unless otherwise stated.

*OA* older adults, *PD* Parkinson’s disease, *MCI* mild cognitive impairment, *n/a* not applicable.

### Model selection

The XGBoost model provided the most accurate predictions, while the simple regression tree was less accurate (Table 2) among the ML models. Moreover, the standard deviation of the RMSE obtained using the XGBoost model was the lowest, showing its robustness to different splits of the data. In the following sections, we describe several modifications to improve the model’s performance and implementation in real-time by eliminating the need for a separate step segmentation process, focusing on the XGBoost model.

**Table 2:** Step length average RMSE and standard deviation of five ML models and one biomechanical model for the test set (recall Table 1, top panel)

Model	LR	RT	SVM	KNN	XGBoost	Inverted Pendulum
**RMSE [cm]**	6.46 ± 0.20	7.43 ± 0.23	6.31 ± 0.30	7.07 ± 0.21	6.08 ± 0.15	20.60 ± 0.77
**ICC (2,1)**	0.89 ± 0.01	0.85 ± 0.01	0.89 ± 0.01	0.86 ± 0.01	0.91 ± 0.003	0.54 ± 0.24

The test set refers to Table 1 top panel. Values are presented as mean ± standard deviation.

*RMSE* root mean square error, *LR* linear regression, *RT* regression tree, *SVM* support vector machine, *KNN* K-nearest neighbors, *XGBoost* extreme gradient boosting, *ICC* intraclass correlation coefficient.

**Fig. 1:**
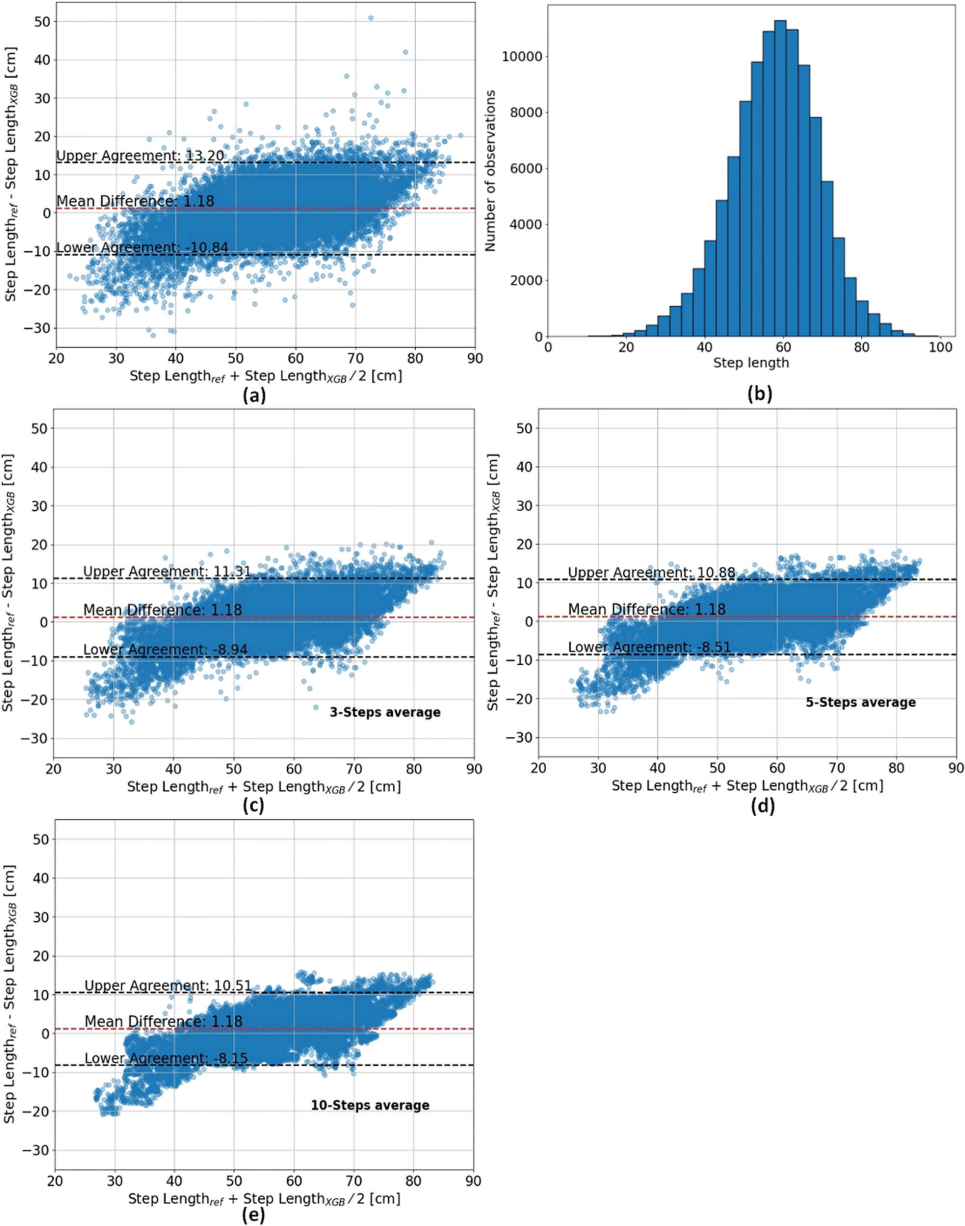
Comparison of step length measurements and XGBoost estimates: Bland-Altman plot and step length distribution. **a**, **c**–**e** Bland-Altman for 1, 3, 5, and 10 steps. The middle line (red) represents the mean difference and the lower and upper lines (black) represent the 95% limits of agreement. **b** Step length distribution.

**Fig. 2:**
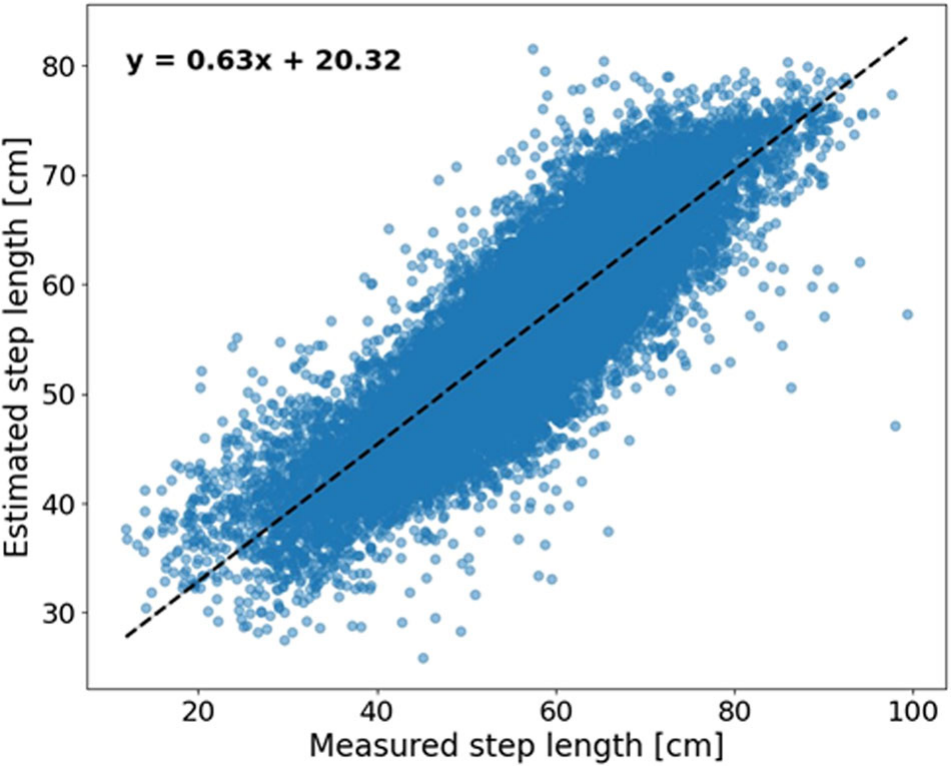
Correlation between the XGBoost estimated step length as a function of the measured step length. The blue dots represent the estimated step length as a function of the measured step length. The dashed black line represents the trend.

The step length histogram is presented in Fig. 1b for the test set (i.e., V-TIME dataset; recall Table 1, top panel). Figure 1a shows the Bland-Altman plot of the estimated step length using the XGBoost model. The 95% limits of agreement between the estimated step length and the measured step length are in the range of −10.84 and 13.20 cm. Moreover, a trend can be observed, suggesting that the model tends to estimate a close to average step length—underestimates large step length and overestimates small step length. Figure 2 presents the regression analysis for the estimated step length. The Pearson correlation coefficient and *R*^2^ are 0.86 and 0.71, respectively, which indicates that there is a strong positive linear correlation between the estimated and the measured step length.

### Averaging technique for improving SLE accuracy

To improve the SLE accuracy, we employed an averaging technique by calculating the mean of estimated and measured step lengths across several consecutive steps. It should be noted that the model is trained on a single-step length, and therefore, the averaging was applied to single-step estimations. The RMSE was reduced to 5.21, 4.98, and 4.79 cm for *n* = 3, 5, and 10, respectively. This decrease signifies a notable improvement (F-statistic = 23.0, *p* value = $4.8* {10}^{-6}$ according to the ANOVA test when comparing no average to the average over 3, 5, and 10 steps). However, it is worth noting that this averaging approach resulted in a loss of the ability to determine step-to-step variability that can also be used as another measure of the subject’s gait. Figure 1c–e present the Bland-Altman plot for the 3, 5, and 10-step averages, respectively. The 95% limits of agreement between the estimated step length and the measured step length decrease when the averaging is applied on a larger number of steps until reaching a range of −8.15 cm to 10.51 cm for the ten-step averaging.

**Fig. 3:**
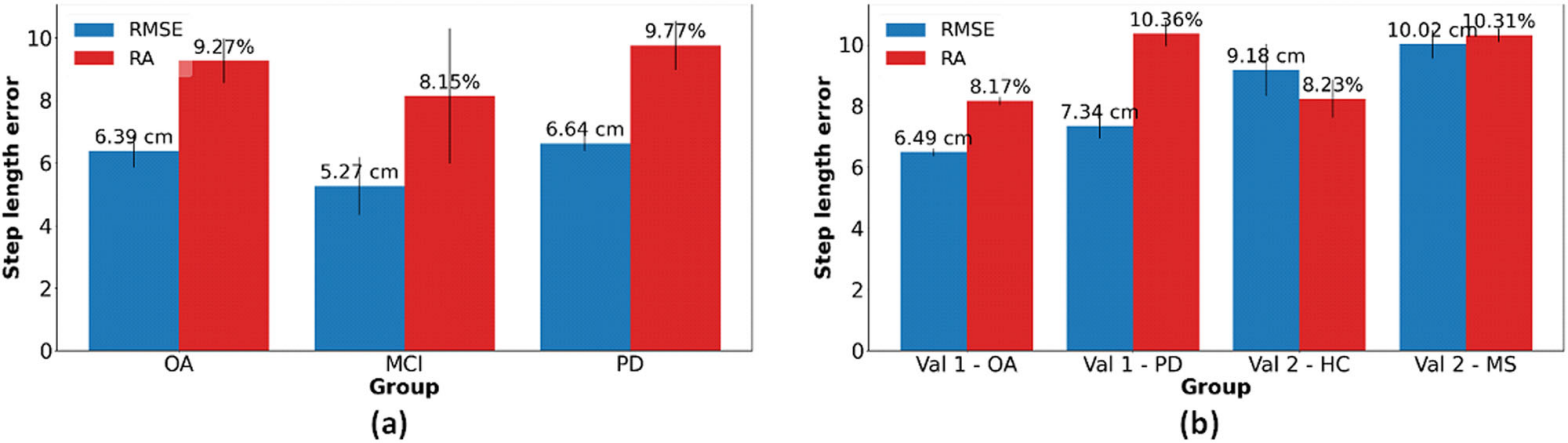
RMSE and RA of different participant groups. **a** Test set. **b** Two validation sets. The blue bars represent the RMSE, and the red bars represent the RA. The error bars represent the standard deviation of the fivefolds. The test set refers to Table 1 top panel (PD, MCI, OA) and the validation sets refer to Table 1 middle and bottom panels (MS, HC, OA, PD).

**Fig. 4:**
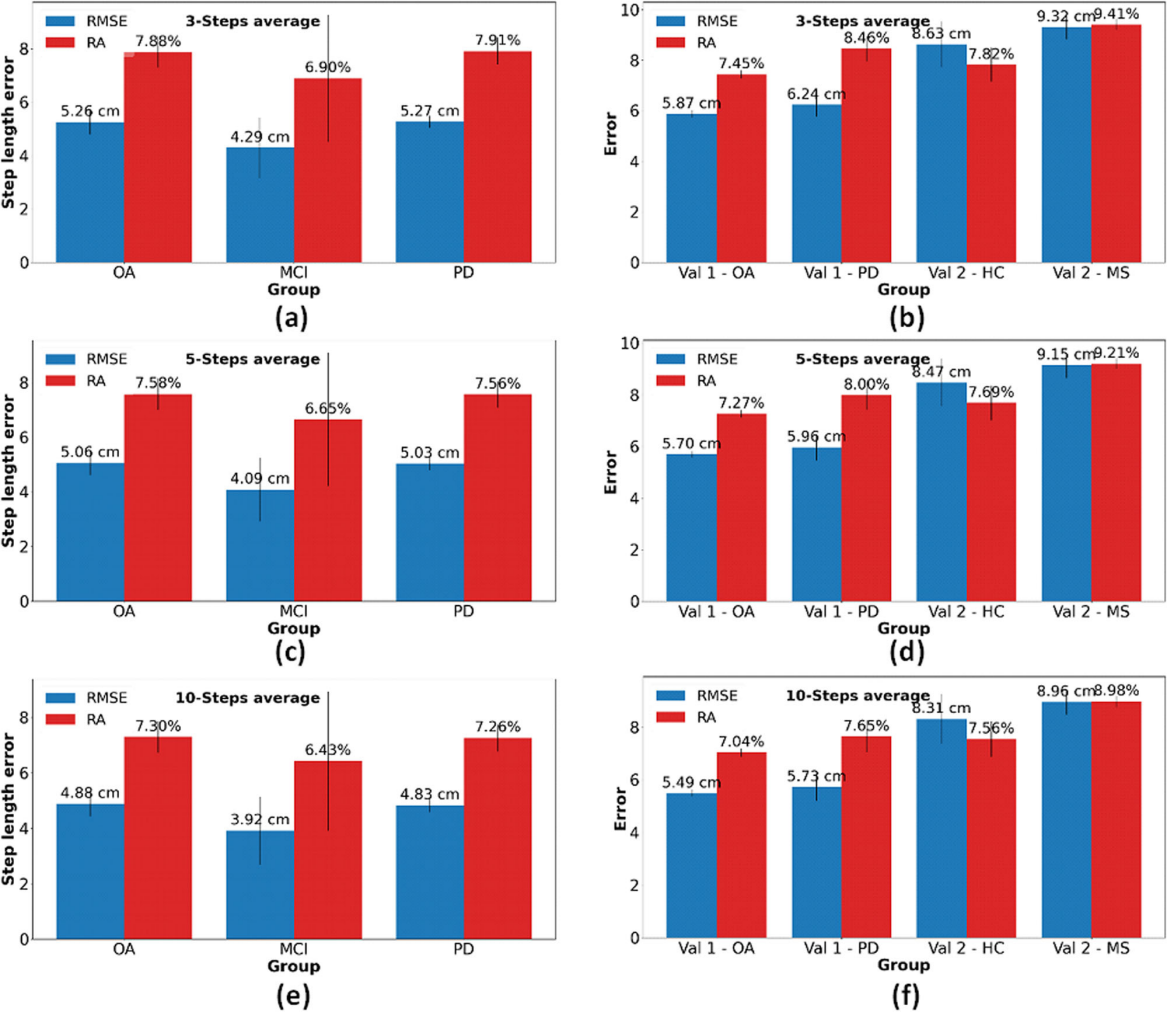
RMSE and RA of different participants groups for n-steps average. **a** Test set (recall Table 1 top panel) with *n* = 3. **b** Validation sets with *n* = 3. **c** Test set with *n* = 5. **d** Validation sets with *n* = 5. **e** Test set with *n* = 10. **f** Validation sets with *n* = 10. The blue bars represent the RMSE and the red bars represent the RA. The error bars represent the standard deviation of the fivefolds. The test set refers to Table 1 top panel and the validation sets refer to the middle and bottom panels of Table 1.

Table 3 and Figs. 3, 4 presents the step length RMSE for each group (PD, MCI, and OA) from the test set and the n-average RMSE where n equals 1, 3, 5, and 10. The RMSE of the PD participants was the highest (6.64 cm), whereas the lowest RMSE was obtained for the MCI participants (5.27 cm), i.e., a relatively large difference, although not statistically different (*t* value = 1.76, *p* value = 0.12) from the OA participants (6.39 cm). When averaging the estimated step length, the RMSE for the MCI group decreased to 3.92 cm. Table 4 presents the step length RMSE for each of several walking conditions. The error for comfortable walking speed was the lowest (5.70 cm) while the RMSE for fast walking speed was the highest (6.72 cm). This finding matches other results, showing that the model is less accurate for extreme values (and less accurate for slow and fast walking).

**Table 3:** Step length RMSE, RA, and intraclass correlations for different groups of participants in the test set (recall Table 1, top panel)

	OA participants (*n* = 81)			MCI participants (*n* = 27)			PD patients (*n* = 149)		
	RMSE [cm]	RA [%]	ICC (2,1)	RMSE [cm]	RA [%]	ICC (2,1)	RMSE [cm]	RA [%]	ICC (2,1)
Single SL	6.39 ± 0.52	9.20 ± 0.61	0.90 ± 0.02	5.27 ± 0.93	8.22 ± 2.16	0.77 ± 0.15	6.64 ± 0.25	9.59 ± 0.48	0.89 ± 0.01
3-average SL	5.26 ± 0.45	7.88 ± 0.57	0.92 ± 0.01	4.29 ± 1.12	6.90 ± 2.38	0.82 ± 0.13	5.27 ± 0.23	7.91 ± 0.50	0.92 ± 0.01
5-average SL	5.06 ± 0.45	7.58 ± 0.58	0.93 ± 0.01	4.09 ± 1.17	6.65 ± 2.44	0.83 ± 0.13	5.03 ± 0.23	7.56 ± 0.49	0.92 ± 0.01
10-average SL	4.88 ± 0.45	7.30 ± 0.57	0.93 ± 0.01	3.92 ± 1.22	6.43 ± 2.51	0.83 ± 0.13	4.83 ± 0.24	7.26 ± 0.48	0.93 ± 0.01

The test set refers to Table 1 top panel. Values are presented as mean ± standard deviation. RMSE Root mean square error. The correlation is the Pearson’s coefficient between the model estimate and the reference value.

*OA* older adults, *PD* Parkinson’s disease, *MCI* mild cognitive impairment, *SL* step length, *RA* relative error.

**Table 4:** Step length RMSE and RA for different gait conditions in the test set (recall Table 1, top panel)

	Usual walking speed		Fast walking speed		Dual tasking Walking	
	RMSE [cm]	RA [%]	RMSE [cm]	RA [%]	RMSE [cm]	RA [%]
Single SL	5.70 ± 0.25	8.50 ± 0.37	6.72 ± 0.35	8.80 ± 0.42	6.26 ± 0.25	10.65 ± 0.45
3-average SL	4.78 ± 0.28	6.96 ± 0.42	5.77 ± 0.35	7.47 ± 0.40	5.24 ± 0.23	8.86 ± 0.39
5-average SL	4.59 ± 0.28	6.64 ± 0.43	5.55 ± 0.35	7.18 ± 0.39	5.00 ± 0.22	8.45 ± 0.38
10-average SL	4.43 ± 0.29	6.36 ± 0.43	5.35 ± 0.34	6.91 ± 0.36	4.78 ± 0.22	8.08 ± 0.36

The test set refers to Table 1 top panel. Values are presented as mean ± standard deviation. The correlation is Pearson’s coefficient.

*RMSE* root mean square error, *RA* relative error.

### Non-Segmented model for real-time implementation

A fundamental step in the process of SLE is step segmentation, in which each straight-line walking segment of a subject is segmented into steps. As an alternative, it is possible to train a model using fixed-size windows. Unlike the previously described model, in this case, the windows do not necessarily contain a whole number of steps. Using this training method does not require step segmentation and, therefore, is more suitable for real-time and real-world (daily living) implementation of SLE since we can estimate the distance/gait speed at each time point. This model will be denoted as the “non-segmented model”. The comparison between the original model and a non-segmented model cannot be performed in terms of step length error because the non-segmented model estimates the traveled distance in a fixed time window rather than steps. Therefore, we compared the gait speed RMSE of the different models. The gait speed RMSE for two models that were trained on a constant walking segment (1 and 5 s) were 12.4 and 11.8 cm/s, respectively. The gait speed RMSE of the original model that was trained on step length was 11.4 cm/s. Although the original model that was trained on a single step provided the lowest gait speed RMSE, the RMSE of the models trained on 5 consecutive seconds and one second of a walking segment were similar.

### Model generalizability on validation datasets

The model generalizability was further tested on the four validation datasets (recall Table 1). Figure 3 illustrates the RMSE and RA values for the test set (Fig. 3a), along with the validation sets (Fig. 3b). Notably, the RMSE was observed to be slightly larger for the validation set 2, due perhaps to the inherently larger step length, while the RA remained relatively consistent across the datasets. The step length RMSE increased for the validation set 1-OA participants. However, the RA decreased. Both RMSE and RA increased for the participants with PD of the first validation set. The second validation set included 102 participants, with younger ages (40.00 ± 11.13 yrs compared to 73.38 ± 7.01 yrs), comprising both healthy and MS participants. The step length RMSE increased for both the healthy control group and the MS group. However, The RA of the second validation set for the healthy control group was smaller than the RA of the original healthy control group and comparable to the MCI group. The RA of the MS participants group was larger than the RA of the original PD group.

## Discussion

The present analyses were based on a relatively large number of subjects (almost 500) with a range of health status and conditions, including healthy older and young adults, and participants with MCI, PD, and MS. Our goal was to accurately estimate the step length from a single IMU, placed on the lower back, using ML models within controlled gait settings, without the need for calibration or the measurement of a subject’s height or weight. We tested several models and found that the XGBoost provided the best result for the test set (RMSE = 6.08 cm for a single step and lower values when averaged over multiple steps).

When comparing the model’s performance to other biomechanical estimators, such as the inverted pendulum model^51^, a large improvement was observed (6.08 vs 20.60 cm). The model presented a strong linear correlation between the estimated and the measured step length. However, the Bland-Altman plot, presented in Fig. 1a, revealed that the developed model tended to underestimate large step lengths while overestimating short step lengths. This observation suggests a systematic bias in the model’s estimations, with a consistent deviation towards smaller step length estimations for longer steps and towards larger step length estimations for shorter steps. While this limitation is a shared challenge among many models and may pose concerns for the precision required in diagnostic settings^52,53^, it may have a comparatively lesser impact as a progression biomarker that often relies more on within-subject changes over time. A progression biomarker based on within-subject changes may still capture disease progression across most step lengths, with potential overestimation only in large steps, which are less common in individuals with neurological disorders, and minimal underestimation in small steps. Still, further research is required to enhance SLE, especially at the extremes of the gait spectrum and for a single-step value.

The averaging technique reduced the RMSE to values lower than 5 cm for 5 steps (4.98 cm) and 10 steps (4.79 cm). The improvement in the RMSE is expected to increase as the number of averaged steps grows, at least up to a certain value. Since we average the label to be predicted (and not the data), the regression task becomes easier as the number of steps increases (the function to predict becomes smoother). In other words, the input data (single step data) remains the same in all of the experiments, while the step length to predict is not the original number that was associated with the single step, but an average of 3, 5, or 10 sequential steps. Thus, it is reasonable that the error decays as the number of steps averaged increases. In the present work, we provided a sense of the decay rate. Although this method decreased the RMSE to achieve our predefined goal (MCID 5 cm), it led to a loss of the ability to study step-to-step variability that can also serve as an important measure of one’s gait. Further work can help to identify the trade-offs between averaging over many steps, a few steps, or not at all. Perhaps, the optimal point may depend on the specific application. Alternatively, for some purposes, it may be helpful to analyze both the average measure and the non-averaged measures, as done in many previous studies using other measurement approaches^1,6,22,23^.

Another finding is that the model’s performance among PD participants is the worst (RMSE = 6.64 ± 0.25 cm), whereas its performance on MCI participants is the best (RMSE = 5.27 ± 0.93 cm). Estimating the step length of PD participants may be more challenging due to the irregularities in their walk, and therefore, we expected that the step length estimation would be the least accurate among the different groups. However, among the three groups who participated in the initial study, the PD participants constituted the largest subgroup, hence the model was trained largely from participants with this group’s walking pattern and therefore we speculated that the model could estimate this group’s step length more accurately. Although the RMSE obtained for the MCI group was the lowest, the standard deviation of the MCI participants was the largest, indicating that there is a relatively large variability in the model’s performance in this subgroup. Along with the low ICC obtained for this group (0.77), this suggests that some participants’ walks in this group were much harder to estimate, likely due to the relatively small number of participants within this subgroup. It is worth noting that while all of the participants in the V-TIME study (the test set) had a history of two or more falls at baseline, falls were much more frequent among the people with PD (19 in 6 months) and were lowest in MCI (2.9 in 6 months; OA: 3.2^54^). In addition, the model’s performance was analyzed for the different gait tests that the participants performed. This analysis was performed to explore potential variations in the model’s performance across different walking patterns, which may inform future studies, especially in uncontrolled environments. The SLE during fast walking was, somewhat surprisingly, the least accurate in terms of RMSE (although the RMSE was still only 6.72 cm for a single step, and this was reduced when averaging over 3, 5, or 10 steps). Conversely, when examining the RA, our model faced the greatest challenges during dual-task walking. Fast walking inherently involves longer step lengths, perhaps leading to larger RMSE values. In contrast, dual tasking typically results in shorter step lengths, which suggests that the model encounters greater difficulty in accurately estimating step length when the individual is engaged in a secondary task, perhaps because walking is typically less regular and more variable in this condition. These findings imply that the model is influenced to some extent by the walking speed, potentially constraining its applicability. This information can be used in the future to improve the model based on the different characteristics and walk types of the desired group. For example, if the participants are known to walk at or near a usual gait speed, the model can be trained only on this dataset, and it can learn only the patterns belonging to this type of walk. Alternatively, one could consider a two-stage model, wherein gait speed or step length is first estimated crudely, and then a more fine-tuned approach is applied to refine the estimate, similar to the approach taken by Byun et al. ^38^.

Our dataset contains a diverse range of groups, including older adults and persons with either MCI or PD, each undergoing several gait tests. Additionally, our assessment included separate validation sets—ONPAR and MS-Watch, yielding consistent outcomes. This result underscores the robustness of the selected features; the differences in the error obtained in the test and validation sets were relatively small. Although the step length RMSE of the second validation set for both groups was larger, the RA was comparable to controls. The reason for that may be due to the different step lengths of the two datasets. The original dataset has a mean step length of 57.72 cm, whereas the second validation set has a mean step length of 73.94 cm. Therefore, the RA may be a more representative measure of error. The first validation set consists of two groups of participants—healthy adults and participants with PD. The RMSE of healthy adults for the first validation set is slightly larger than the value obtained on the test set test set (6.49 cm compared to 6.39 cm). When comparing the RA, the model performed better on the first validation set (8.17% compared to 9.27%). We note that the RMSE for all of the groups in the two validation sets were above the MCID, when examining the estimation error for a single step, and specifically the results for the second validation set. As mentioned above, the RMSE can be biased when representing the error for larger step length and this might contribute to this outcome. In addition, the second validation set includes young and healthy adults, with different gait characteristics. The model was trained on a completely different population and, therefore, it is possible that training it on an even more diverse dataset would yield better outcomes.

Another modification that we explored was training the model on an arbitrary walking segment, which simplified the data processing pipeline. The gait speed RMSE that we obtained for a 5-s segment was not lower than the gait speed RMSE for one step, but it was comparable, showing that this preprocessing step could be eliminated without a large increase in the gait speed RMSE. The gait speed RMSE that was obtained for a single-step estimation in our study was larger (11.4 ${cm}/\sec$) compared to the one achieved by Byun et al. ^38^ (6.81 ${cm}/\sec$). However, although the result obtained by Byun et al. ^38^ is impressive, their model requires using demographic and anthropometric features which makes this method less convenient. The study presented by Sabatini et al. ^55^ received a similar result to the one obtained by Byun et al. ^38^. However, while Sabatini et al. ^55^ utilized two wearable devices, positioned at the pelvis and shank, our study employed a single device placed on the lower back. Furthermore, Sabatini et al. ^55^ involved a cohort of young and healthy participants, in contrast to our model, which underwent testing across various age groups and conditions, including PD, MS, and healthy subjects. Wang et al. ^56^ employed a geometrical model utilizing four IMUs, tested on ten healthy subjects and five with gait impairment. Our model exhibits enhanced accuracy, particularly for subjects with gait impairment, compared to Wang et al. ^56^. Additionally, our model’s utilization of a single IMU located on the lower back enhances practical convenience. Kose et al. ^34^ obtained an excellent result of less than 3% error for step length but it was only tested in nine young and healthy participants. A study employing a deep neural network achieved an impressive step length mean absolute error of 0.2396 cm but utilized a limited dataset with only four participants. Furthermore, the method’s reliance on five IMUs attached to the participants likely limits its practical applicability^57^. Moreover, in the specific context of participants with MS, the study presented by Motl et al. ^58^ included 51 participants with MS and demonstrated slightly inferior performance compared to our model (12 cm/sec). This highlights the robust generalizability of our proposed model across diverse participant profiles and health conditions.

The recent study presented by Micó-Amigo et al. ^40^, which may be considered the current state-of-the-art, reviewed the performance of several step length estimators when they were applied to 108 participants with various health conditions (including PD and MS). The best estimator achieved stride length absolute errors of 15 and 17 cm for the healthy adult group and PD group, respectively. The absolute error that we obtained for stride length for both groups is 12 cm. In addition, the intraclass correlation coefficients that were obtained were also lower than the intraclass correlation coefficient that we obtained (0.58–0.60 vs 0.89–0.90). From this perspective, the XGBoost model outperforms the state-of-the-art model. However, non-straightline trajectories and walking at everyday activities were also included in the previous study. On the other hand, the datasets that we used included numerous participants with different conditions but were only collected in laboratory settings in a controlled environment. Therefore, our model still needs to be tested on more realistic walking patterns to validate our method compared to state-of-the-art methods. In addition, when applied to real-world walking, it needs to be combined with an algorithm that detects turning. It is also important to recall that our model underwent rigorous testing on two additional and separate datasets, yielding consistent results. In contrast, prior studies^36–39^ relied on methodologies like 5-fold cross-validation, leave-one-out, and train-test splits, which may limit generalizability.

Overall, our results show that the described XGBoost model can be used as an accurate step length estimator, even in people with relatively impaired gait like that seen among older adults, people with PD or MS, a capability that is currently lacking in most estimators. Locating wearable devices on the lower back is relatively convenient for patients and offers practical advantages^59^. It remains discreet (out of sight, out of mind) and does not necessitate specific footwear, while still providing reliable acceleration and gyroscope signals that can be employed in a machine-learning model. The simplicity of this method makes it a potential candidate for a single-device solution in clinical settings, especially in controlled testing environments. Future studies are needed to optimize the model in real-world and uncontrolled settings. In addition, as discussed above, the model’s performance decreases when reaching relatively large or small step lengths and still needs to be further improved, although the errors are still relatively small at larger and smaller step lengths (recall Fig. 2). Moreover, a very recent study by Baudendistel et al. ^47^ reported a step length MCID of 3.6 cm in participants with PD, slightly lower than that RMSE achieved in the present study. Nonetheless, reaching the current target of an error of 5 cm is an important step forward, enhancing the ability to use a single sensor to estimate step length and, ultimately, to bring wearable devices closer to routine clinical use, potentially enabling more accurate monitoring of patients in settings that are more relevant to them.

## Methods

**Fig. 5:**
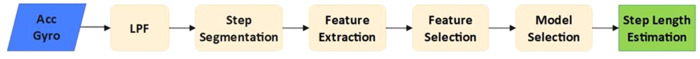
SLE flow chart. Schematic description of the proposed algorithmic steps.

The methodology employed in this research involves the assembly of deidentified database based on previously collected data, preprocessing, step segmentation, feature extraction and selection, and a model that is able to estimate step length. Figure 5 illustrates the process, with detailed explanations provided in the subsequent sections. The secondary analysis was conducted in compliance with all relevant ethical regulations, including the Declaration of Helsinki, as approved by the human studies committee of the Tel Aviv Sourasky Medical Center. In the original data collection studies, written informed consent was obtained from all human participants.

### Database assembly

**Fig. 6:**
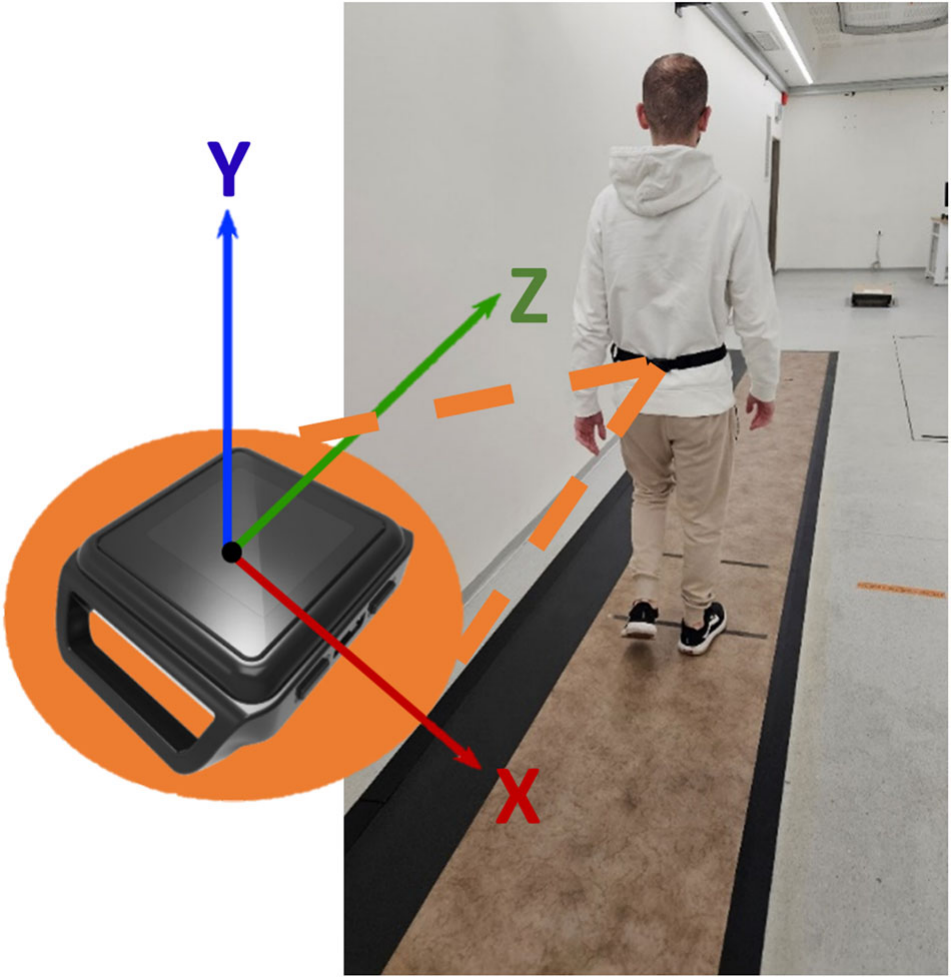
Experimental setup. Subject walking over the Zeno Walkway with the IMU placed on the lower back. Orientation of the IMU axes is illustrated.

The data for training, testing, and validating the models were taken from three projects. Data of the first project were obtained from a previously described V-Time study^48^: 149 patients with PD (age 71.1 ± 6.1 yrs, Movement Disorders Society Unified Parkinson’s Disease Rating Scale (MDS-UPDRS) score 63 ± 21), 27 people with mild cognitive impairment (age 77.5 ± 6.3 yrs, Montreal Cognitive Assessment (MoCA) score 21.6 ± 3.9), and 81 older adults (76.9 ± 6.2 yrs). All participants had a history of 2 or more falls. Participants performed three 1-min gait tests in the same order: (1) comfortable speed, (2) fast speed, and (3) while performing an additional cognitive task (counting aloud backward and subtracting by 3 s). During the testing, subjects wore an Opal sensor on the lower back, recording 3D acceleration and 3D gyroscope signals at 128 Hz (APDM Inc, Portland, OR, USA). As shown in Fig. 6, the X, Y, and Z axes align with the mediolateral, vertical, and anterior-posterior directions, respectively. The authors affirm that human research participants provided informed consent for publication of the image in Fig. 6. The subjects walked over a Zeno Walkway Gait Analysis System (Protokinetics LLC, Havertown, PA) with a length of 7.92 m, which served as the gold-standard measure of step length (and gait speed). Participants were assessed four times during the study—before, after, 1 month after, and 6 months after the intervention (the testing order was the same at each time point). A total of 83,569 steps were evaluated.

In the second project, named ONPAR, data was collected in a similar way to the methodology outlined earlier, involving the use of Opal sensors and Zeno Walkway. It includes participants with similar ages (68.35 ± 7.77 yrs): 75 patients with PD (age 67.98 ± 7.25 yrs, MDS-UPDRS total score 31 ± 12), and 38 healthy adults (69.07 ± 8.71 yrs). The third project, named MS-Watch, included a younger group of participants (40.0 ± 11.1 yrs): 61 patients with MS (age 42.0 ± 11.3 yrs, expanded disability status scale, EDSS 2.24 ± 1.57, disease duration 10.12 ± 8.80 yrs), and 41 healthy adults (37.0 ± 10.3 yrs). The V-Time dataset was used for training and testing of the model, using a fivefold cross-validation, and is referred to as the test set. The ONPAR and MS datasets were used for the assessment of the model and are referred to as validation set 1 and validation set 2, respectively. This approach differs from previous studies that employed a leave-one-out or utilized only a k-fold without a distinct validation set.

### Signal preprocessing

The preprocessing phase involved two key steps. First, the linear acceleration and angular velocity signals were low-pass filtered using an FIR filter with a cutoff frequency of 20 Hz. This step aimed to remove high-frequency noise and unwanted artifacts. Then, the signal was segmented into single steps using a step segmentation algorithm described in ref. ^40^, which is based on the vertical acceleration signal. The step segmentation process enabled subsequent feature extraction and machine-learning algorithms to operate on distinct step intervals with varying lengths. Finally, the Opal-segmented steps were synchronized to the Zeno Walkway measurements by minimizing the time difference between each detected step from the two sensors.

### Feature extraction and selection

Features were extracted as described in the Supplementary information. To remove irrelevant data and to reduce the overfitting error in the examined ML models, it is necessary to use a feature selection method that eliminates those features and keeps only meaningful features. We used a stepwise feature selection method^60^ in which, in each iteration, the features that contributed most to the model’s accuracy were added. This process was performed on a small portion of the data and validated using cross-validation to ensure that the selected features are robust. Thirty-four features were selected as the most important (described in Supplementary Table 1), including the FFT coefficients of the acceleration signal, the acceleration’s magnitude energy, and the second integration of the X and Y axes of the acceleration.

### Model selection and validation

We tested several traditional ML models, including linear regression, regression tree, SVM, and KNN, due to their simplicity and their computational efficiency. In addition, the XGBoost model was tested, consisting of gradient-boosted decision trees known for their state-of-the-art results on many tabular datasets^61^. To further assess the performance and versatility of our models, we also evaluated an inverted pendulum model^51,62,63^. This biomechanical model estimates the step length using the changes of the vertical position of the center of mass during gait and was included to investigate whether it outperformed the ML models in estimating step length accurately. We used a fivefold cross-validation to provide a reliable estimate of the model’s performance. The hyper-parameters of the model were optimized for each fold using the fold training set and according to the hyper-parameters range specified in Supplementary Table 2. The V-TIME dataset was used for training and testing while keeping each participant either in the training set or validation set for each fold. The ONPAR and MS-Watch datasets were only used for validation (and not in training). Gait speed was subsequently determined using the estimated step length and duration. In addition, two completely independent datasets, named ONPAR and MS-Watch, were used to further evaluate the generalizability of the model.

### Modifications

Several enhancements were made to improve SLE accuracy using the lower back-mounted IMU. Firstly, an averaging technique was employed in which the mean of estimated and measured step lengths across several consecutive steps was calculated. This approach aimed to minimize the effect of irregularities in individual step estimation, by averaging the estimations and the reference values and not the features themselves. On the other hand, it is important to note that variability assessment may be compromised when averaging multiple steps. Additionally, an innovative training method was employed, where a model was trained on an arbitrary walking segment that could consist of more than one step. The walking segment length was fixed to a constant time segment and the model was trained with two different lengths —1 and 5-s segments, to find an appropriate segment length. Each gait sequence was randomly sampled at various time points to create several gait segments, with the number of segments determined by the length of the straight-line walking segment. Rather than restricting the training process to predefined step-based segments, our approach allowed for the inclusion of a varying time segment, extending beyond the confines of a single step. This approach eliminated the need for explicit segmentation of individual steps, thus simplifying the data processing pipeline.

### Statistical analysis

As a measure of accuracy, we used the root mean square error (RMSE) of the step length, the RMSE of the gait speed, and the relative error (RA) of the step length. Intraclass correlation coefficient (ICC (2,1))^64^ was calculated to assess the association between the SLE and the step length measurements by the Zeno Walkway. Based on ICC estimates, values less than 0.5, between 0.5 and 0.75, between 0.76 and 0.9, and greater than 0.90 were deemed to be indicative of poor, moderate, good, and excellent reliability, respectively^65^. Additionally, we employed Bland-Altman analysis and assessed the Limits of Agreement (LoA) to further examine the agreement between the estimated step lengths and those measured by the Zeno Walkway. *R*² was also determined to measure how well the estimated step length matches the reference values, showing how much of the variation in step lengths is captured by our model. Pearson’s correlation coefficient greater than 0.8 was considered a strong correlation^66^. To determine the effect of the averaging technique on the RMSE, we performed a one-way analysis of variance (ANOVA) with a *P* value of 0.05.

### Reporting summary

Further information on research design is available in the Nature Research Reporting Summary linked to this article.

### Supplementary information

Supplementary material

Reporting Summary

## Data Availability

The data analyzed in this study will be made available upon reasonable request and as allowed by human study committees.

## Code availability

The underlying code for this study will be available at https://github.com/assafzadka/XGB-SLE/tree/main.
