# Supplementary material for: A wearable sensor and machine learning estimate step length in older adults and patients with neurological disorders
Source: NPJ Digit Med. 2024 May 25;7:142. doi: 10.1038/s41746-024-01136-2 (PMC11127966; doi:10.1038/s41746-024-01136-2)
Supplement: Supplementary file 1 — Supplementary material [file 41746_2024_1136_MOESM1_ESM.docx]

**Supplementary material**

The following set of features, a total of 111, were extracted for all step segments from both 3D linear acceleration and angular velocity component signals:

- Step frequency.
- The minimum, maximum, mean, and standard deviation for all three components, and of the relevant absolute value.
- First and second integration all three signal components and the relevant absolute value.
- Correlation coefficients between all signal components.
- Energy of all three signal components , and the relevant absolute value.
- First six coefficients (representing frequencies 0, 2, 4, 6, 8, and 10 Hz) of the FFT of All three signal components energy, and the relevant absolute value.

**Supplementary Table 1. Overview of the selected features**

| **Feature** | **Description** |
| --- | --- |
| ‘ACC min 1’ | Minimal value of the X component of the linear acceleration |
| ‘ACC min 3’ | Minimal value of the Z component of the linear acceleration |
| ‘ACC min mag’ | Minimal value of the absolute value of the linear acceleration |
| ‘ACC max mag’ | Maximal value of the absolute value of the linear acceleration |
| ‘ACC std 1’ | Standard deviation of the X component of the linear acceleration |
| ‘ACC std 2’ | Standard deviation of the Y component of the linear acceleration |
| ‘ACC std 3’ | Standard deviation of the Z component of the linear acceleration |
| ‘ACC v mag’ | First integration of the absolute value of the linear acceleration |
| ‘ACC s 1’ | Second integration of the X component of the linear acceleration |
| ‘ACC s 2’ | Second integration of the Y component of the linear acceleration |
| ‘ACC s mag’ | Second integration of the absolute value of the linear acceleration |
| ‘Gyro mean 2’ | Mean value of the Y component of the angular acceleration |
| ‘Gyro std mag’ | Standard deviation of the absolute value of the angular acceleration |
| ‘Gyro s 2’ | Second integration of the Y component of the angular acceleration |
| ‘COR_COEF Gyro 1 2’ | Correlation coefficients between the X and Y components of the angular acceleration |
| ‘total pow ACC mag’ | Energy of the absolute value of the linear acceleration |
| ‘StepFreqancy’ | Step frequency |
| ‘FFT_COEF ACC 2 1’ | First FFT coefficient of the Y component of the linear acceleration |
| ‘FFT_COEF Gyro 1 1’ | First FFT coefficient of the X component of the angular acceleration |
| ‘FFT_COEF Gyro 2 1’ | First FFT coefficient of the Y component of the angular acceleration |
| ‘FFT_COEF ACC 1 2’ | Second FFT coefficient of the X component of the linear acceleration |
| ‘FFT_COEF ACC 3 2’ | Second FFT coefficient of the Z component of the linear acceleration |
| ‘FFT_COEF Gyro 2 2’ | Second FFT coefficient of the Y component of the angular acceleration |
| ‘FFT_COEF ACC mag 3’ | Third FFT coefficient of the absolute value of the linear acceleration |
| ‘FFT_COEF ACC 2 3’ | Third FFT coefficient of the Y component of the linear acceleration |
| ‘FFT_COEF ACC 3 3’ | Third FFT coefficient of the Z component of the linear acceleration |
| ‘FFT_COEF Gyro mag 3’ | Third FFT coefficient of the absolute value of the angular acceleration |
| ‘FFT_COEF ACC mag 4’ | Fourth FFT coefficient of the absolute value of the linear acceleration |
| ‘FFT_COEF ACC mag 5’ | Fifth FFT coefficient of the absolute value of the linear acceleration |
| ‘FFT_COEF ACC 2 5’ | Fifth FFT coefficient of the Y component of the linear acceleration |
| ‘FFT_COEF ACC mag 6’ | Sixth FFT coefficient of the absolute value of the linear acceleration |
| ‘FFT_COEF ACC 2 6’ | Sixth FFT coefficient of the Y component of the linear acceleration |
| ‘FFT_COEF ACC 3 6’ | Sixth FFT coefficient of the Z component of the linear acceleration |
| ‘FFT_COEF Gyro 1 6’ | Sixth FFT coefficient of the Z component of the angular acceleration |

**Supplementary Table 2. XGBoost hyper-parameters**

| **Hyper-parameter** | **Range** |
| --- | --- |
| learning_rate | [0.05, 0.1] |
| gamma | [0, 0.5] |
| max_depth | [3, 6] |
| min_child_weight | [1, 10] |
