# Supplementary material for: A wearable sensor and machine learning estimate step length in older adults and patients with neurological disorders
Source: NPJ Digit Med. 2024 May 25;7:142. doi: 10.1038/s41746-024-01136-2 (PMC11127966; doi:10.1038/s41746-024-01136-2)
Supplement: Supplementary file 2 — Reporting Summary [file 41746_2024_1136_MOESM2_ESM.pdf]

nature portfolio

Corresponding author(s): Jeffrey Hausdorff

Last updated by author(s): Apr 22, 2024

Reporting Summary

Nature Portfolio wishes to improve the reproducibility of the work that we publish. This form provides structure for consistency and transparency in reporting. For further information on Nature Portfolio policies, see our Editorial Policies and the Editorial Policy Checklist.

Statistics

For all statistical analyses, confirm that the following items are present in the figure legend, table legend, main text, or Methods section.

n/a	Confirmed	
☐	☒	The exact sample size (*n*) for each experimental group/condition, given as a discrete number and unit of measurement
☐	☒	A statement on whether measurements were taken from distinct samples or whether the same sample was measured repeatedly
☐	☒	The statistical test(s) used AND whether they are one- or two-sided<br>*Only common tests should be described solely by name; describe more complex techniques in the Methods section.*
☒	☐	A description of all covariates tested
☒	☐	A description of any assumptions or corrections, such as tests of normality and adjustment for multiple comparisons
☐	☒	A full description of the statistical parameters including central tendency (e.g. means) or other basic estimates (e.g. regression coefficient) AND variation (e.g. standard deviation) or associated estimates of uncertainty (e.g. confidence intervals)
☐	☒	For null hypothesis testing, the test statistic (e.g. *F*, *t*, *r*) with confidence intervals, effect sizes, degrees of freedom and *P* value noted<br>*Give P values as exact values whenever suitable.*
☒	☐	For Bayesian analysis, information on the choice of priors and Markov chain Monte Carlo settings
☒	☐	For hierarchical and complex designs, identification of the appropriate level for tests and full reporting of outcomes
☐	☒	Estimates of effect sizes (e.g. Cohen's *d*, Pearson's *r*), indicating how they were calculated

Our web collection on statistics for biologists contains articles on many of the points above.

Software and code

Policy information about availability of computer code

Data collection	No software was used for the data collection.
Data analysis	The underlying code for this study will be available at: https://github.com/assafzadka/XGB-SLE/tree/main.<br>The model was developed using the Python software and all the data analysis was performed using Python.

For manuscripts utilizing custom algorithms or software that are central to the research but not yet described in published literature, software must be made available to editors and reviewers. We strongly encourage code deposition in a community repository (e.g. GitHub). See the Nature Portfolio guidelines for submitting code & software for further information.

Data

Policy information about availability of data

All manuscripts must include a data availability statement. This statement should provide the following information, where applicable:

- Accession codes, unique identifiers, or web links for publicly available datasets
- A description of any restrictions on data availability
- For clinical datasets or third party data, please ensure that the statement adheres to our policy

The data analyzed in this study will be made available upon reasonable request and as allowed by human study committees.

Research involving human participants, their data, or biological material

Policy information about studies with human participants or human data. See also policy information about sex, gender (identity/presentation), and sexual orientation and race, ethnicity and racism.

Reporting on sex and gender	The data includes male and female sex participants. Sex was not considered in the study design and we did not preforme any analysis involving it, since this was not the goal of the study. Overall 472 participants took part in the study (248 females, 224 males). Based on previous studies, there is no reason to believe that a machine learning model's ability to estimate step length will depend on sex or gender.
Reporting on race, ethnicity, or other socially relevant groupings	Race and ethnicity were not reported or considered in the study. Based on previous studies, there is no reason to believe that a machine learning model's ability to estimate step length will depend on race, ethnicity, or socially relevant groupings.
Population characteristics	The V-Time dataset includes 149 patients with PD (age 71.1±6.1 yrs, Movement Disorders Society Unified Parkinson's Disease Rating Scale (MDS-UPDRS) score 63±21), 27 people with mild cognitive impairment (age 77.5±6.3 yrs, Montreal Cognitive Assessment (MoCA) score 21.6±3.9), and 81 older adults (76.9±6.2 yrs). The ONPAR dataset includes participants with similar ages (68.35±7.77 yrs): 75 patients with PD (age 67.98±7.25 yrs, MDS-UPDRS total score 31±12), and 38 healthy adults (69.07 ±8.71 yrs). The third project, named MS-Watch, included a younger group of participants (40.0±11.1 yrs): 61 patients with MS (age 42.0±11.3 yrs, Expanded Disability Status Scale, EDSS 2.24±1.57, disease duration 10.12±8.80 yrs), and 41 healthy adults (37.0±10.3 yrs).
Recruitment	The present work was the secondary analyses of data originally collected for other purposes. The recruitment strategy used in the original studies are described in the original studies. Briefly, study participants who met relevant inclusion and exclusion criteria were recruited from local clinics, referral, and word-of-mouth. Since the goal of the present work was to estimate step length, and since a subjects with a range of health and disease status were included we anticipate that recruitment details will not impact estimation success.
Ethics oversight	The study was approved by the human studies committee of the Tel Aviv Sourasky Medical Center.

Note that full information on the approval of the study protocol must also be provided in the manuscript.

Field-specific reporting

Please select the one below that is the best fit for your research. If you are not sure, read the appropriate sections before making your selection.

☒ Life sciences ☐ Behavioural & social sciences ☐ Ecological, evolutionary & environmental sciences

For a reference copy of the document with all sections, see nature.com/documents/nr-reporting-summary-flat.pdf

Life sciences study design

All studies must disclose on these points even when the disclosure is negative.

Sample size	Sample size was of the original studies was determined by each of those studies. We used all available data in the present study.
Data exclusions	Data was excluded in any case of misalignment between the steps times detected by the Opal IMUs and the Zeno Walkway Gait Analysis System.
Replication	We used a 5-fold cross-validation to provide a reliable estimate of the model's performance. When using the same split (using the exact seed in Python) the results can be fully replicated. Different splits can lead to a small deviance from the reported result.
Randomization	For the V-time dataset, we used 5-fold cross-validation keeping each participant either in the training set or validation set for each fold. This step ensures that no data leakage will affect the results. The ONPAR and MS-Watch datasets were only used for validation (and not in training) and therefore this data was not randomized.
Blinding	Blinding was not relevant to our study since the goal was to develop a model that can preform SLE for different groups pf participants.

Reporting for specific materials, systems and methods

We require information from authors about some types of materials, experimental systems and methods used in many studies. Here, indicate whether each material, system or method listed is relevant to your study. If you are not sure if a list item applies to your research, read the appropriate section before selecting a response.

Materials & experimental systems

n/a	Involved in the study
☒	☐ Antibodies
☒	☐ Eukaryotic cell lines
☒	☐ Palaeontology and archaeology
☒	☐ Animals and other organisms
☒	☐ Clinical data
☒	☐ Dual use research of concern
☒	☐ Plants

Methods

n/a	Involved in the study
☒	☐ ChIP-seq
☒	☐ Flow cytometry
☒	☐ MRI-based neuroimaging

Plants

Seed stocks	*Report on the source of all seed stocks or other plant material used. If applicable, state the seed stock centre and catalogue number. If plant specimens were collected from the field, describe the collection location, date and sampling procedures.*
Novel plant genotypes	*Describe the methods by which all novel plant genotypes were produced. This includes those generated by transgenic approaches, gene editing, chemical/radiation-based mutagenesis and hybridization. For transgenic lines, describe the transformation method, the number of independent lines analyzed and the generation upon which experiments were performed. For gene-edited lines, describe the editor used, the endogenous sequence targeted for editing, the targeting guide RNA sequence (if applicable) and how the editor was applied.*
Authentication	*Describe any authentication procedures for each seed stock used or novel genotype generated. Describe any experiments used to assess the effect of a mutation and, where applicable, how potential secondary effects (e.g. second site T-DNA insertions, mosiacism, off-target gene editing) were examined.*
